# How does mortality compare between different countries/regions of birth for the population of England and Wales, 2007 to 2021? A descriptive, observational study

**DOI:** 10.1177/01410768251377564

**Published:** 2025-12-12

**Authors:** Lucinda Hiam, Jon Minton, Rachel Burns, Robert W Aldridge

**Affiliations:** 1 School of Geography and the Environment, University of Oxford, Oxford, UK; 2 School of Social & Political Sciences, University of Glasgow, Glasgow, UK; 3 Collaborative Centre for Inclusion Health, University College London, London, UK; 4 Department of Health Metric Sciences, University of Washington, Seattle, USA

**Keywords:** Health inequalities, migrant health, mortality, public health, standardised mortality ratios (SMRs)

## Abstract

**Objectives:**

To examine all-cause mortality differences among migrants from different countries/regions compared with native-born populations in England and Wales from 2007 to 2021 and assess whether migrant mortality patterns converge with or diverge from native-born trends over the study period.

**Design:**

Descriptive, observational study analysing mortality trends over a 15-year period.

**Setting:**

England and Wales, using national mortality records and Census data.

**Participants:**

The study included all recorded deaths in England and Wales from 2007 to 2021, stratified by sex and country/region of birth.

**Main outcome measures:**

European age-standardised rates (EASRs) and standardised mortality ratios (SMRs) for all-cause mortality, with and without COVID-19-related deaths. Linear regressions were used to assess mortality trends over time.

**Results:**

Mortality patterns varied significantly by country/region of birth. While most migrant groups had lower mortality rates than the native-born population at the beginning of the study period, this advantage declined for many in recent years. Migrants from Ireland, Scotland and Northern Ireland exhibited consistently worse mortality outcomes. Excluding COVID-19 deaths, 14 out of 19 migrant groups retained a mortality advantage, though trends indicate substantial heterogeneity. Some migrant groups, particularly from North and Central America and parts of Europe, showed improving mortality rates, whereas others, such as those from Bangladesh, converged towards native-born mortality levels.

**Conclusions:**

Despite lacking data on individual-level factors (e.g. duration of residence, socio-economic status and co-morbidities), this national-level study demonstrates important trends in migrant mortality. The findings highlight the urgent need for improved data to capture migration-related variables and enable a deeper understanding of the drivers behind observed trends. The decline in migrant mortality advantage over time for some groups highlights the importance of monitoring structural health inequalities and can inform targeted public health policies and more granular future research.

## Introduction

Between the 2011 and 2021 censuses the population of England & Wales (E&W) that were born outside of the United Kingdom (hereon referred to as ‘migrants’) increased by 2.5 million, making up 10 million (16.8%) of the population by 2021.^1^ This period saw a growing number of policies embedding immigration control within public services, including the National Health Service (NHS), alongside shifts in mobility due to Brexit and the COVID-19 pandemic (although Freedom of Movement did not officially end until Brexit’s implementation on 11 December 2020).^2,3^ While overall numbers of migrants increased, there was a decrease in European Union (EU) migration, and an increase in non-EU migration.^4^

Migrants often face barriers to healthcare, discrimination, xenophobia and associated health harms.^5,6^ Despite these challenges, evidence demonstrates that migrants from low- and middle-income countries experience lower mortality rates than native-born populations in high-income countries (HICs),^7,8^ with migrants contributing positively to life expectancy.^9–11^ In the USA, immigration has mitigated some of the recent life expectancy deterioration and stagnation.^10^ This ‘migrant mortality advantage’ is linked to the selective migration of healthier individuals.^12,13^ However, exceptions exist, with mortality advantages and disadvantages varying by disease and country/region of birth.^14–17^ For example, migrants from Ireland living in E&W^17^ and those born in Finland living in Sweden^9^ show higher mortality than the native-born population (or a health *disadvantage*).

The mortality advantage can diminish over time,^12,18,19^ a form of ‘levelling down’, linked to negative acculturation in the country of residence (described as the ‘selection-acculturation hypothesis’)^12^ and socio-economic challenges, including barriers to healthcare,^20^ adoption of adverse health behaviours, poor housing and employment conditions, and the negative impacts of racism and xenophobia.^5^ These social determinants of health may partially explain the worse outcomes some migrants experienced during the COVID-19 pandemic.^21^

**Figure 1.**
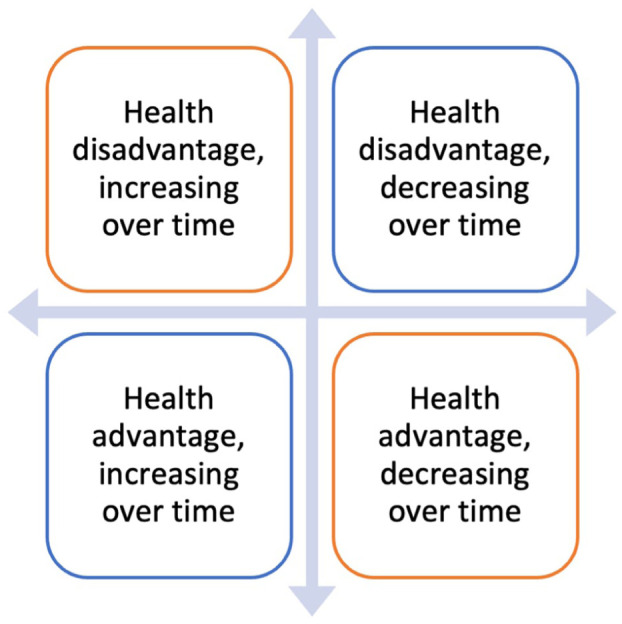
Four possible outcomes per country/region of birth when compared with the England & Wales-born population from 2007 to 2021.

Given the recent political and demographic shifts, this study examines two questions: (1) How does all-cause mortality vary among migrants from different countries/regions compared with native-born populations in E&W? (2) Are migrant mortality patterns converging with or diverging from native-born trends? We propose four scenarios for migrant health over time: a health disadvantage that (a) increases or (b) decreases over the years examined, or a health advantage that (c) increases or (d) decreases (Figure 1). While previous studies have explored some of these dynamics, they have often focused on shorter timeframes or specific groups. This study extends the analysis to 15 years (2007 to 2021), using annual mortality data by age, sex and country/region of birth for E&W to provide a more comprehensive understanding of long-term trends. This repeated cross-sectional approach is widely used in national mortality surveillance and while it cannot assess individual-level causality, it enables the detection of structural changes in mortality patterns over time. Our data do not include the length of time in E&W since migration, and thus the impact of negative acculturation cannot be assessed.

## Methods

### Study design and setting

A descriptive, observational study of the population of E&W hereafter from 2007 to 2021 was carried out. The exposure was identified as the country/region of birth, as identified on the death certificate, and the outcome was annual mortality from all causes over the 15-year period. Notably, this is a repeated cross-sectional analysis, which observes population-level mortality trends over time without tracking the same individuals.

### Data sources

This study used two data sources. Data were requested from the Office for National Statistics (ONS) for the number of deaths by country/region of birth, 5-year age band, sex and ICD-10 chapter from 2007 to 2021, based on vital registration data of deaths. The mortality data are subject to disclosure control and therefore not publicly available, but reported within the ONS guidance.^22^ Second, for the total population, we used data from the last two censuses in 2011 and 2021, linearly interpolating for the years in between.^23^ While this introduces some uncertainty, it is a commonly accepted method for national-level estimates and is used by statistical agencies such as the ONS and Human Mortality Database. These population estimates form the denominators for calculating age-standardised rates and standardised mortality ratios (SMRs).

### Inclusion and exclusion criteria

The countries/regions of birth included were decided by (a) the available data from the ONS and (b) the ‘matching’ across the deaths and total population datasets (outlined below). Two countries/regions were excluded in line with disclosure control (‘Africa NOS’ and ‘Asia NOS’) due to low counts.^22,24^ Data cleaning was carried out to ensure consistent groupings of countries/regions of birth were applied between the datasets of the mortality and population data, collapsing populations to lowest common denominators where possible. Where a match was not possible, certain data that were disaggregated in one dataset, but not both, were excluded. For example, we were not able to disaggregate ‘new EU’ (i.e. those countries that joined after 2004) from total EU (i.e. all EU countries, including those that joined after 2004) in all datasets, and thus ‘new EU’ was excluded as an individual category, with EU as total included. In total, 20 countries/regions of birth were included (19 plus E&W born). Appendix I lists the countries included where a region is specified. People aged 20 years and over were included and standardised to the European age-standardised population (EASP), as outlined below. Those under 20 years old were excluded due to the low counts.

### Analysis

Data preparation and analyses were carried out using R version 4.4.2.^25^ First, we calculated the annual European age-standardised rates (EASRs) for all-cause mortality by the 20 country/regions of birth, including E&W, and sex, based on the 2013 EASP,^26^ with 95% confidence intervals from 2007 to 2021. Recognising the impact of COVID-19 on mortality data from 2020 onwards, we then calculate the EASRs for all-cause mortality excluding deaths from COVID-19. As we are focused on the trends over time from 2007 to 2021, we used the data without deaths from COVID-19 for these analyses. We conducted a COVID-19-specific mortality analysis separately.^27^ Second, we used the EASRs for all-cause mortality excluding COVID-19 to produce SMRs using the corresponding rates for each sex born in E&W as the reference population (i.e. SMR = 1) via the PHEindicatormethods package in R.^28^ Analyses were conducted separately by sex, and 95% confidence intervals calculated using Byar’s method in R.^29^ Third, to further assess the relationship between SMRs for migrants and non-migrants over time, we used linear regressions to estimate the direction of trends in EASRs by the available country/region of birth compared with the E&W reference population between 2007 and 2021, reported with *p* values. The code for performing the analyses is available on request (note the mortality data are not available as these are subject to disclosure control).

### Ethical approval

Ethical approval was not sought as the data are aggregate and anonymised. Data were handled subject to the disclosure agreement with ONS.

## Results

### European age-standardised rates by sex and country/region of birth, 2007 to 2021

#### All-cause mortality including COVID-19, 2007 to 2021

**Figure 2.**
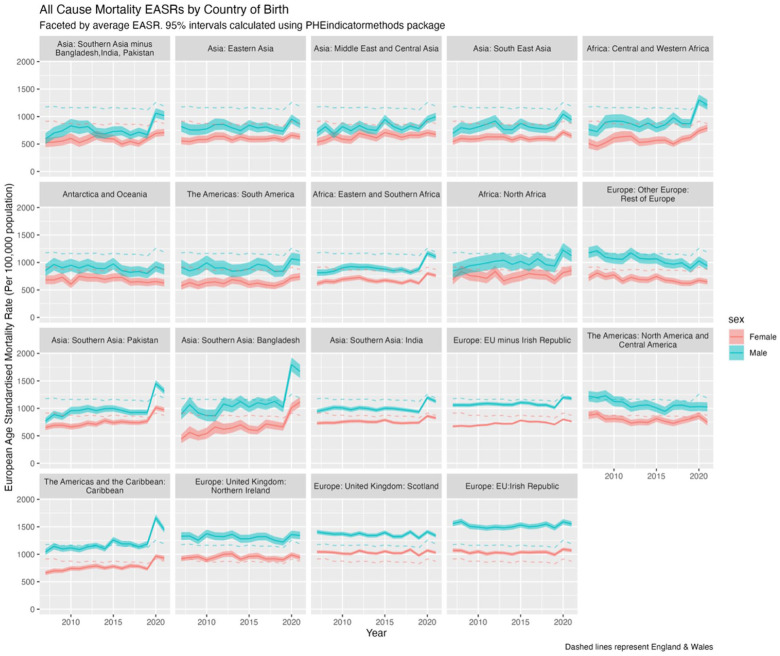
EASRs (per 100,000 population) for all-cause mortality (including COVID-19) by sex and country/region of birth for the population of England & Wales, 2007 to 2021, with 95% confidence intervals (shaded). Dashed lines represent the reference population of males and females born in England & Wales.

Figure 2 presents the EASRs for all-cause mortality by sex and country/region of birth for migrants from 2007 to 2021, with reference populations of those born in E&W shown as dashed lines. Ten of the 19 migrant sub-populations had lower EASRs than the E&W-born population, including in 2020 and 2021. However, for some groups, initial mortality advantages were lost by 2020–2021. For example, males from Central and Western Africa and both sexes from Pakistan and Bangladesh had initially lower mortality than E&W-born population, but by 2020 and 2021 their rates became higher. Bangladesh, in particular, experienced a notable 50% mortality increase from 2019 (Appendix I, Figure S1).

Migrants from Northern Ireland, Scotland and the Irish Republic had consistently higher EASRs than the native-born population throughout 2007 to 2021, without the pandemic-related uptick seen in other groups. Males from the Caribbean had similar EASRs to the native-born population until 2020–2021, when their mortality rates increased, while females from the Caribbean, initially lower, also converged with native-born rates. The varying impacts of COVID-19 mortality by the same regions/countries of birth are explored elsewhere.^27^ Figure 2 shows diverging trends, with some migrant populations (e.g. North & Central America; European populations) showing improving mortality rates i.e. diverging from E&W-born up until 2020, while others, for example, Bangladesh, converged toward E&W reference populations.

#### All-cause mortality excluding COVID-19, 2007 to 2021

**Figure 3.**
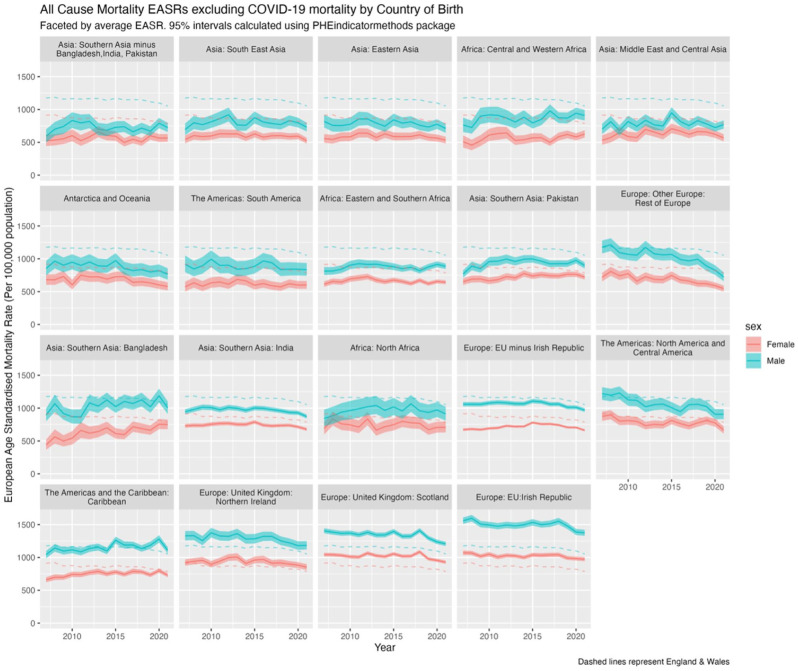
EASRs (per 100,000 population) for all-cause mortality excluding COVID-19 in 2020 and 2020-2021 by sex and country/region of birth for the population of England & Wales, 2007 to 2021, with 95% confidence intervals (shaded). Dashed lines represent the reference population of males and females born in England & Wales.

To explore the impact of COVID-19 on mortality trends, Figure 3 excludes COVID-19 deaths from the analysis in 2020 and 2021. Without COVID-19, 14 of the 19 migrant sub-populations had consistently lower EASRs than the native-born population. When compared with Figure 2, this suggests that increases in 2020 and 2021 for Central & Western Africa and Pakistan appear largely due to COVID-19 mortality, whereas for Bangladesh and the Caribbean, COVID-19 appears only a partial explanation.

### Annual standardised mortality ratios by sex and country/region of birth, 2007 to 2021

#### All-cause mortality excluding COVID-19, 2007 to 2021

To examine trends over time, we focus on mortality excluding COVID-19 due to the unprecedented and unusual behaviour of the virus in 2020 and 2021. Results for all-cause mortality SMRs, *including* COVID-19, can be found in Appendix I, Figure S2.

**Figure 4.**
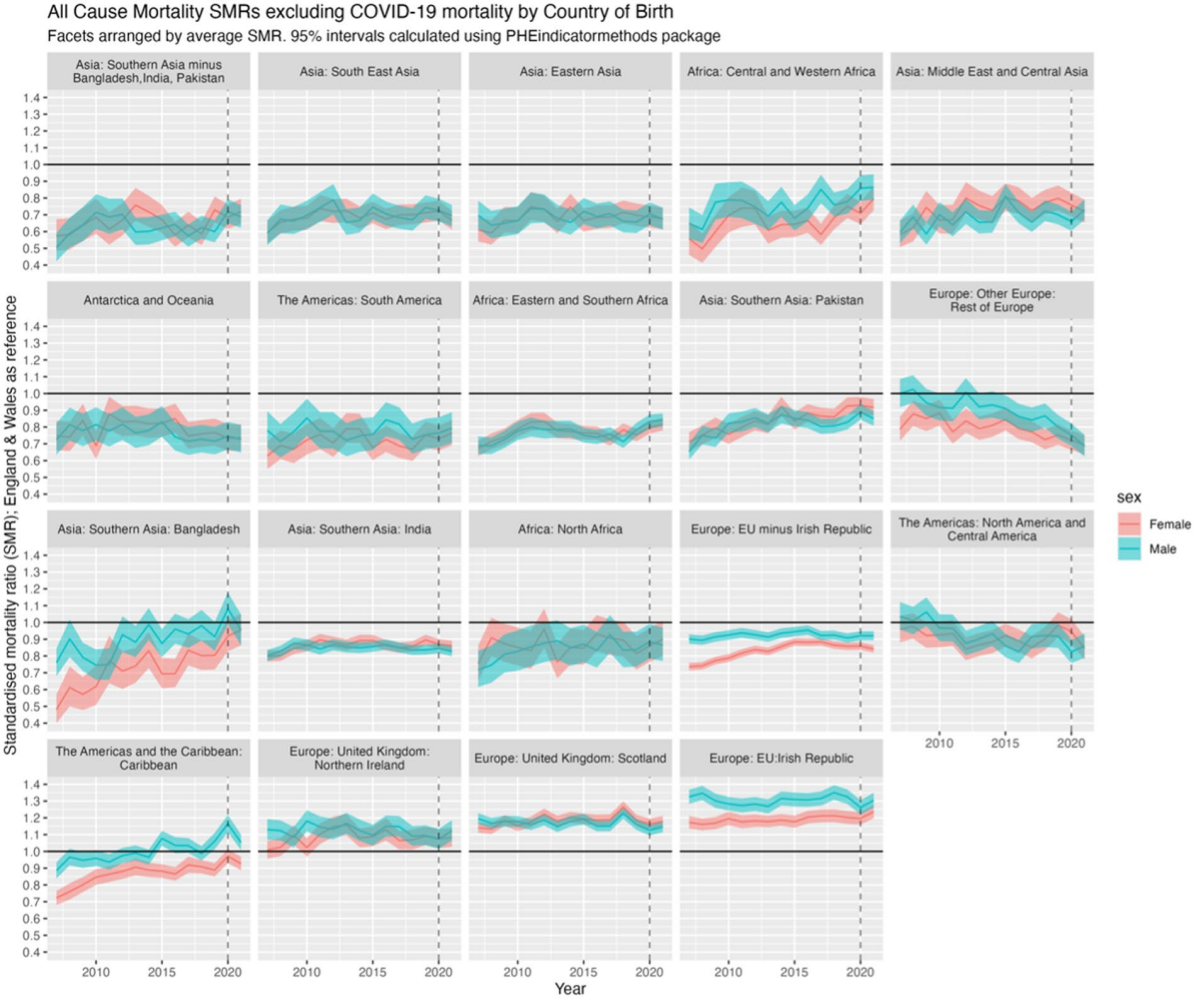
SMRs for all-cause mortality excluding COVID-19 in 2020 and 2021 by sex and country/region of birth for migrants compared with non-migrants for the population of England & Wales, 2007 to 2021, with 95% confidence intervals (shaded). Horizontal **black** line at 1.0 represents the SMR of the reference population of males and females born in England & Wales.

Figure 4 presents SMRs for all-cause mortality (excluding COVID-19) for the 19 countries/regions of birth compared with the native-born population (SMR = 1; shown with **black** horizontal line). The facets (subfigures) are arranged by average SMR, from most advantageous, that is, lowest SMR (<1) compared with E&W (Southern Asia minus Bangladesh, India, Pakistan) at the top left to most disadvantageous, that is, highest SMR (>1) compared with E&W (Irish Republic) at the bottom right. A SMR >1 indicates higher mortality than the E&W-born population, and a SMR less than 1 a lower mortality. Trends align with Figures 3 and 4, with some of the trends in mortality improving and diverging from E&W seen more clearly (prior to 2020 and 2021), for example, North & Central America and Rest of Europe, and converging of, for example, Bangladesh.

### Regression of trends in SMRs between 2007 and 2021

**Figure 5.**
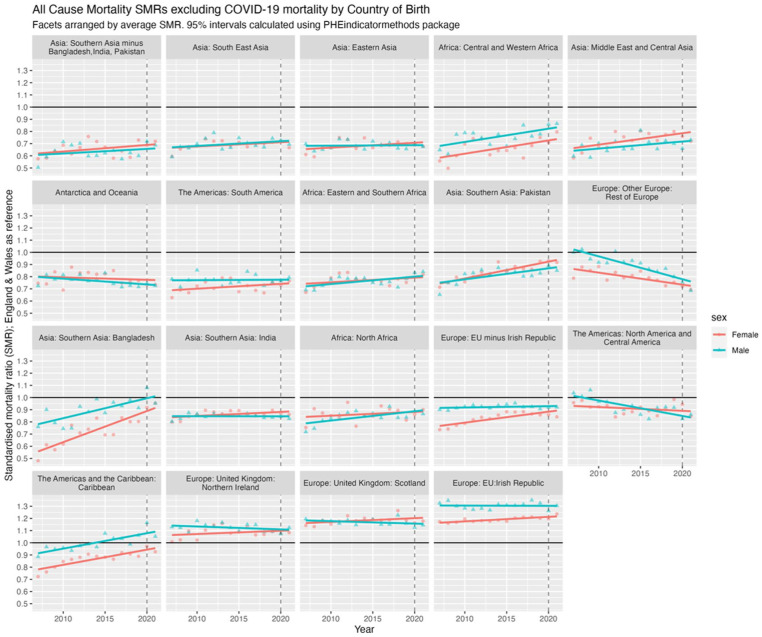
All-cause mortality SMRs excluding COVID-19 by country/region of birth and sex for migrants in England & Wales, 2007 to 2021, with linear regression lines. SMR for E&W shown as black line (=1). SMR <1, an upward trending line suggests a mortality advantage that is worsening, that is, converging with E&W; if SMR >1, a downward trend suggests a mortality disadvantage that is improving, that is, converging with E&W. Thus, further information is required for interpretation (see Table S1, S2, Figures S3, S4 in Appendix).

The facet positions in Figure 4 that show the relative mortality (dis)advantage – that is, better or worse mortality than the E&W-born population – of each country/region of birth on average between 2007 and 2021, but do not tell us whether such relative (dis)advantages have changed over time. Figure 5 shows the linear regression of SMRs over time, indicating whether mortality advantages or disadvantages have increased or decreased. If the average SMR between 2007 and 2021 is below 1, then an upwards trend over time indicates the relative advantage compared with E&W-born has been decreasing (or worsening) over time, converging with E&W mortality; if the SMR is on average above 1, then the downwards trends indicate the disadvantage has been decreasing over time; and so forth. However, without the mean SMR, these results are difficult to interpret, as it is not clear whether there is a mortality advantage (SMR < 1) that is improving/worsening, or a mortality disadvantage (SMR > 1) that is improving/worsening. Thus, we present the slope coefficients for SMR trends and mean SMR against the slope coefficient for each/country region and sex in Section 2.2 of the Appendix.

**Figure 6.**
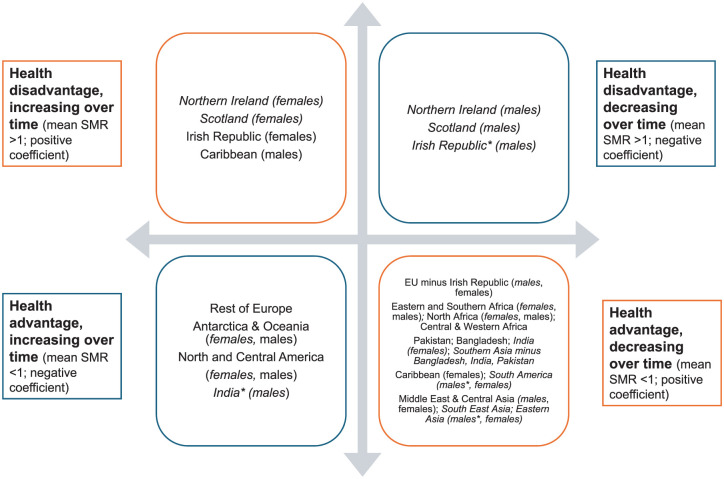
Summary of linear regression of mean SMR from 2007 to 2021 and slope coefficient with England & Wales-born as reference population. Results are for both sexes except where sex differences were present, which are then indicated in parentheses; with those in italics not significant at the 0.05 level. Full results in Section 2.2, Appendix. The asterisk * indicates coefficient 0.000 to three decimal places i.e. trend may be considered stable rather than increasing or decreasing.

Figure 6 categorises countries/regions into four broad trends: decreasing/worsening advantage, increasing/improving advantage, worsening disadvantage, and improving disadvantage. Most countries/regions of birth from 2007 to 2021 have seen the mortality advantage seen in 2007 decrease closer to that of non-migrants in later years.

The 2 × 2 typology presented here highlights systematic heterogeneity in migrant health trends and challenges assumptions of a uniform migrant experience. While statistical significance is noted (Appendix 2.2), the primary role of regression analysis is to conceptualise these variations rather than test specific hypotheses. Countries/regions without significant results (*p* > 0.05) are denoted in italics.

## Discussion

### Main findings of this study

We used ONS data to examine mortality differences by country/region of birth among the population of E&W from 2007 to 2021. This typology-based approach improves the interpretability of complex population trends and offers a structured framework for understanding which groups are gaining, maintaining or losing a mortality advantage. We explored how all-cause mortality varied among migrants from different countries/regions compared with native-born individuals and whether these mortality patterns converged towards or diverged from the native-born mortality over time. We found that 10/19 countries/regions of birth had lower EASRs than the reference E&W-born population over the study period, including during the COVID-19 pandemic in 2020 and 2021 (the divergence is explored in a separate paper).^27^ Excluding COVID-19 deaths, 14/19 migrant groups had lower mortality than the native-born population. Exceptions included migrants from the Irish Republic, Scotland, Northern Ireland and the Americas & the Caribbean, with notable sex differences.

Over time, most countries/regions fell into the ‘health advantage, decreasing’ category, meaning any advantage found in 2007 was diminishing, or absent, by 2021. Three regions (rest of Europe (non-EU), Antarctica & Oceania and North & Central America) saw a mortality advantage that increased over time, diverging further from E&W-born populations.

### What this study adds to the existing literature

#### What is already known on this topic

As discussed in the introduction, migrants in HICs often exhibit a mortality advantage compared with native-born populations,^7,8^ but this advantage can diminish over time due to socio-economic conditions, healthcare access barriers, negative acculturation and the negative impacts of racism and xenophobia.^5,6,12,18,19^ Previous studies have examined specific migrant groups in both E&W and other settings,^9,15,17,24,30^ but up-to-date exploration of long-term trends across multiple countries/regions of birth in E&W following the demographic and political changes outlined remain underexplored.

#### What this study adds

Using 15 years of mortality data in E&W, this study confirms evidence of the healthy migrant effect but shows it has weakened in recent years for most groups. The heterogeneity of migrants is evident. For example, migrants from Ireland, Scotland and Northern Ireland did not exhibit a mortality advantage, consistent with existing research on neighbouring countries in Europe.^9,17^ These findings suggest further exploration is needed on factors such as socio-economic conditions and health behaviours that could influence mortality patterns. Conversely, the three regions that saw a migrant mortality advantage that has increased over time suggest some groups may be benefitting from persistent protective factors. Sex differences in those from the Caribbean, Scotland, Northern Ireland and Ireland also warrant further study.

### Limitations of this study

This study has several limitations, largely shaped by the nature of the available data. Migration-disaggregated national datasets are limited, and the absence of certain variables constrains interpretation. First, we lacked data on duration of residence, essential for examining the selection-acculturation hypothesis. As a result, the changes in mortality advantage/disadvantage over time, summarised in Figure 6, can neither support nor refute this hypothesis.^7,12,18^ The likely bias from this is that our results may be over-estimating mortality rates for more recent migrants, who have had fewer years of negative acculturation and are still benefitting from the healthy selection bias, and under-estimating them for longer-term migrants, potentially subject to negative acculturation.

Second, population denominators for intercensal years were estimated using linear interpolation between the 2011 and 2021 censuses. While standard practice, this method does not capture year-to-year demographic shifts within the decade, such as annual migration flows, composition changes, or emigration, introducing uncertainty and potentially over- or under-estimating mortality rates, particularly for more mobile groups.

Third, the broad grouping of countries/regions may mask important inter- and intra-regional differences, likely under-estimating heterogeneity and obscuring particularly advantaged or disadvantaged sub-groups.

Fourth, we lacked individual-level data on confounders such as health co-morbidities or socio-economic position, limiting our ability to adjust for these factors or fully explain between-group differences. This likely results in some overestimation of the migrant mortality advantage if healthier or more advantaged individuals are overrepresented among migrants.

Fifth, the focus on adults aged 20 years and over and the exclusion of COVID-19 mortality may miss some differences in younger age groups and understate the pandemic’s impact on migrant mortality patterns.

Sixth, we could not directly assess racial or ethnic disparities, which are known to significantly shape health outcomes through structural racism, xenophobia and systemic inequalities.^5^ This likely underestimates the impact of these structural factors on migrant health.

Finally, as the mortality of E&W nationally has worsened since 2010,^31^ convergence of migrant mortality towards native-born levels may partly reflect a deterioration in native-born health, rather than improvements among migrants. Interpreting convergence as a positive trend should therefore be approached with caution.

### Areas for future research

These findings open many avenues for future research. First, cause-specific analyses are needed to unpack the mechanisms behind the observed mortality trends. Disaggregating by cause of death will help identify whether changes are driven by, for example, chronic disease, external causes, or infectious disease, but must be interpreted in light of variation across age, sex and country/region of birth. Second, the development or linkage of individual-level datasets, including information on time since migration, socio-economic position and co-morbidities, is essential to assess the validity of the ‘selection-acculturation’ hypothesis and move from descriptive to causal analysis. Third, further research should investigate how structural factors, such as racism, xenophobia and immigration policies, interact with social determinants of health to shape migrant mortality. Previous research has shown that hostile environment policies, including NHS charging regulations, can have detrimental health impacts.^20,32,33^ Notably, Scotland, Northern Ireland and Ireland, which had worse mortality than E&W, would not be subject to NHS charging regulations, suggesting a complex interplay of influencing factors, likely including the role of race. Racism is a fundamental determinant of health, creating structural barriers that lead to poorer health outcomes through limited healthcare access, environmental hazards and increased psychosocial stress.^5^ The specific impacts of racism and xenophobia on migrant health are beyond the scope and design of this paper, and warrant further exploration in future research.

Fourth, identifying factors contributing to lower mortality in some migrant groups – such as dietary habits, social networks and educational status – could offer valuable insights for improving native-born population health. Finally, comparative studies across HICs with similar migrant populations could test the generalisability of these findings and inform international policy.

### Implications for public health practice and policy

Most migrant groups have better health than the native-born population, but this advantage has reduced over the time examined. While we cannot infer individual-level trajectories or causation from this repeated cross-sectional design, the observed patterns offer a valuable signal of population-level change. As the study does not include time since migration, we cannot say this change is linked to negative acculturation, but we can comment on the change in population-level data at each year studied.

The findings underline the need for targeted, community-led public health interventions to, for example, harness health benefits, where found, and to address the challenges migrants may face in areas such as accessing healthcare and poor housing. They also suggest the need to monitor how structural factors such as immigration policies, institutional racism or access to healthcare may contribute to the erosion of these advantages. Without understanding and addressing these underlying factors, strategies and interventions to mitigate migrant health disparities are unlikely to succeed.

## Conclusion

Migrants make up a significant and growing part of the population of England & Wales. Our findings contribute to existing research by demonstrating substantial variation in mortality among migrant groups. While most groups had a health advantage in the initial years of the study period, this advantage diminished by the end of the period for many.

This study, while not longitudinal, offers a rare national-level view of long-term mortality trends by country of birth. It highlights key areas for public health action and future investigation, particularly the need to link individual-level data to understand the pathways underpinning observed changes. These findings emphasise the complex and challenging interplay between migration and health. Although we cannot attribute the observed convergence to negative acculturation due to the study design, the patterns are consistent with this phenomenon, reinforcing the need for targeted, community-led public health interventions to improve migrant health. Further research is needed to determine if – and why – the migrant mortality advantage diminishes with increasing time since migration. Such insights are vital to enable policymakers and healthcare workers to address these factors and improve outcomes for the whole population.

## Supplemental Material

sj-docx-1-jrs-10.1177_01410768251377564 – Supplemental material for How does mortality compare between different countries/regions of birth for the population of England and Wales, 2007 to 2021? A descriptive, observational study Supplemental material, sj-docx-1-jrs-10.1177_01410768251377564 for How does mortality compare between different countries/regions of birth for the population of England and Wales, 2007 to 2021? A descriptive, observational study by Lucinda Hiam, Jon Minton, Rachel Burns and Robert W Aldridge in Journal of the Royal Society of Medicine

sj-xlsx-2-jrs-10.1177_01410768251377564 – Supplemental material for How does mortality compare between different countries/regions of birth for the population of England and Wales, 2007 to 2021? A descriptive, observational study Supplemental material, sj-xlsx-2-jrs-10.1177_01410768251377564 for How does mortality compare between different countries/regions of birth for the population of England and Wales, 2007 to 2021? A descriptive, observational study by Lucinda Hiam, Jon Minton, Rachel Burns and Robert W Aldridge in Journal of the Royal Society of Medicine

## Declarations

**Data availability:** The mortality data are subject to disclosure control and, therefore, not available. Census data are publicly accessible online. Code available on request to the authors.

**Ethics approval:** The study uses anonymised, aggregate data and ethical approval was not required.

**Contributorship:** LH conceived the idea for the paper. JM carried out all analyses with input and review from LH, RB and RWA. LH drafted the manuscript with significant input from JM, RB and RWA. All authors approved the final draft.

**Provenance:** Not commissioned; peer-reviewed by Frank Lichtenberg, Zipporah Iheozor-Ejiofor, and Julie Morris.
