# Supplementary material for: How does mortality compare between different countries/regions of birth for the population of England and Wales, 2007 to 2021? A descriptive, observational study
Source: J R Soc Med. 2025 Dec 12;119(1):14–24. doi: 10.1177/01410768251377564 (PMC12700778; doi:10.1177/01410768251377564)
Supplement: sj-docx-1-jrs-10.1177_01410768251377564 – Supplemental material for How does mortality compare between different countries/regions of birth for the population of England and Wales, 2007 to 2021? A descriptive, observational study [file sj-docx-1-jrs-10.1177_01410768251377564.docx]

Appendix I: Methods and Results

[1. Countries included by Region 2](#_Toc162383173)

[2. Supplementary Results 8](#_Toc162383174)

[2.1. Annual Standardised Mortality Ratios by sex and country/region of birth, 2007-2021 8](#_Toc162383175)

[All-cause mortality 8](#_Toc162383176)

[2.2. Regression of SMRs relationship 9](#_Toc162383177)

[2.3. Slope coefficients for SMR trends by country/region of birth 10](#_Toc162383178)

# Countries included by Region

| **Country/region category** | **Country** |
| --- | --- |
| Africa: Central and Western Africa | Angola |
|  | Benin |
|  | Burkina Faso |
|  | Cameroon |
|  | Central African Republic |
|  | Chad |
|  | Congo |
|  | Cote d'Ivoire |
|  | Democratic Republic of the Congo |
|  | Equatorial Guinea |
|  | Gabon |
|  | Gambia |
|  | Ghana |
|  | Guinea |
|  | Guinea-Bissau |
|  | Liberia |
|  | Mali |
|  | Niger |
|  | Nigeria |
|  | Sao Tome and Principe |
|  | Senegal |
|  | Sierra Leone |
|  | Togo |
|  | Zambia |
|  | Zimbabwe |
| Africa: Eastern and Southern Africa | Botswana |
|  | Burundi |
|  | Comoros |
|  | Djibouti |
|  | Eritrea |
|  | Ethiopia |
|  | Kenya |
|  | Lesotho |
|  | Madagascar |
|  | Malawi |
|  | Mauritius |
|  | Mayotte |
|  | Mozambique |
|  | Namibia |
|  | Reunion |
|  | Rwanda |
|  | Saint Helena |
|  | Seychelles |
|  | Somalia |
|  | South Africa |
|  | Swaziland |
|  | Uganda |
|  | United Republic of Tanzania |
| Africa: North Africa | Algeria |
|  | Egypt |
|  | Libyan Arab Jamahiriya |
|  | Mauritania |
|  | Morocco |
|  | Sudan |
|  | Tunisia |
|  | Western Sahara |
| Antarctica and Oceania | American Samoa |
|  | Antarctica |
|  | Australia |
|  | Bouvet Island |
|  | British Indian Ocean Territory |
|  | Christmas Island |
|  | Cocos (Keeling) Islands |
|  | Cook Islands |
|  | Fiji |
|  | French Polynesia |
|  | French Southern and Antarctic Lands |
|  | Heard Island and McDonald Islands |
|  | Kiribati |
|  | Marshall Islands |
|  | Micronesia, Federated States of |
|  | Nauru |
|  | New Caledonia |
|  | New Zealand |
|  | Niue |
|  | Norfolk Island |
|  | Palau |
|  | Papua New Guinea |
|  | Pitcairn Islands |
|  | Samoa |
|  | Solomon Islands |
|  | South Georgia South Sandwich Islands |
|  | Tokelau |
|  | Tonga |
|  | Tuvalu |
|  | United States Minor Outlying Islands |
|  | Vanuatu |
|  | Wallis and Futuna Islands |
| Asia: Eastern Asia | China |
|  | Hong Kong |
|  | Japan |
|  | Korea, Democratic People's Republic of |
|  | Korea, Republic of |
|  | Macau |
|  | Mongolia |
|  | Taiwan |
| Asia: Middle East and Central Asia | Armenia |
|  | Azerbaijan |
|  | Bahrain |
|  | Georgia |
|  | Iran (Islamic Republic of) |
|  | Iraq |
|  | Israel |
|  | Jordan |
|  | Kazakhstan |
|  | Kuwait |
|  | Kyrgyzstan |
|  | Lebanon |
|  | Oman |
|  | Palestine |
|  | Qatar |
|  | Saudi Arabia |
|  | Syrian Arab Republic |
|  | Tajikistan |
|  | Turkey |
|  | Turkmenistan |
|  | United Arab Emirates |
|  | Uzbekistan |
|  | Yemen |
| Asia: South East Asia | Brunei Darussalam |
|  | Burma |
|  | Cambodia |
|  | Indonesia |
|  | Lao People's Democratic Republic |
|  | Malaysia |
|  | Maldives |
|  | Philippines |
|  | Singapore |
|  | Thailand |
|  | Timor-Leste |
|  | Viet Nam |
| Asia: Southern Asia minus Bangladesh, India, Pakistan | Afghanistan |
|  | Bhutan |
|  | Nepal |
|  | Sri Lanka |
| Asia: Southern Asia: Bangladesh | Bangladesh |
| Asia: Southern Asia: India | India |
| Asia: Southern Asia: Pakistan | Pakistan |
| Europe: EU minus Irish Republic | Albania |
|  | Austria |
|  | Belgium |
|  | Bulgaria |
|  | Croatia |
|  | Cyprus |
|  | Czech Republic |
|  | Denmark |
|  | Estonia |
|  | Finland |
|  | France |
|  | Germany |
|  | Greece |
|  | Greenland |
|  | Hungary |
|  | Italy |
|  | Latvia |
|  | Lithuania |
|  | Luxembourg |
|  | Malta |
|  | Netherlands |
|  | Poland |
|  | Portugal |
|  | Romania |
|  | Slovakia |
|  | Slovenia |
|  | Spain |
|  | Sweden |
| Europe: EU: Irish Republic | Ireland |
| Europe: Other Europe: Rest of Europe | Åland Islands |
|  | Andorra |
|  | Belarus |
|  | Bosnia and Herzegovina |
|  | Faroe Islands |
|  | Gibraltar |
|  | Guernsey |
|  | Holy See (Vatican City) |
|  | Iceland |
|  | Isle of Man |
|  | Jersey |
|  | Liechtenstein |
|  | Monaco |
|  | Montenegro |
|  | Norway |
|  | Republic of Moldova |
|  | Russia |
|  | San Marino |
|  | Serbia |
|  | Svalbard |
|  | Switzerland |
|  | The former Yugoslav Republic of Macedonia |
|  | Ukraine |
| Europe: United Kingdom: England & Wales | England |
|  | Wales |
| Europe: United Kingdom: Northern Ireland | Northern Island |
| Europe: United Kingdom: Scotland | Scotland |
| The Americas and the Caribbean: Caribbean | Anguilla |
|  | Antigua and Barbuda |
|  | Aruba |
|  | Bahamas |
|  | Barbados |
|  | Bermuda |
|  | British Virgin Islands |
|  | Cape Verde |
|  | Cayman Islands |
|  | Cuba |
|  | Dominica |
|  | Dominican Republic |
|  | Grenada |
|  | Guam |
|  | Haiti |
|  | Jamaica |
|  | Montserrat |
|  | Netherlands Antilles |
|  | Northern Mariana Islands |
|  | Puerto Rico |
|  | Saint Barthelemy |
|  | Saint Kitts and Nevis |
|  | Saint Lucia |
|  | Saint Martin |
|  | Saint Vincent and the Grenadines |
|  | Trinidad and Tobago |
|  | Turks and Caicos Islands |
|  | United States Virgin Islands |
| The Americas: North America and Central America | Belize |
|  | Canada |
|  | Costa Rica |
|  | El Salvador |
|  | Guatemala |
|  | Honduras |
|  | Mexico |
|  | Nicaragua |
|  | Panama |
|  | Saint Pierre and Miquelon |
|  | United States |
| The Americas: South America | Argentina |
|  | Bolivia |
|  | Brazil |
|  | Chile |
|  | Colombia |
|  | Ecuador |
|  | Falkland Islands (Malvinas) |
|  | French Guiana |
|  | Guadeloupe |
|  | Guyana |
|  | Martinique |
|  | Paraguay |
|  | Peru |
|  | Suriname |
|  | Uruguay |
|  | Venezuela |

# Supplementary Results

## Change in all-cause mortality from 2019 to 2020 and 2021, Bangladesh

Figure S1 shows the 2020 and 2021 all-cause mortality EASR values by sex for those born in Bangladesh as a proportion of the 2019 values. This is over 1.50 for both 2020 and 2021, demonstrating at 50% increase in mortality from the pre-COVID years.


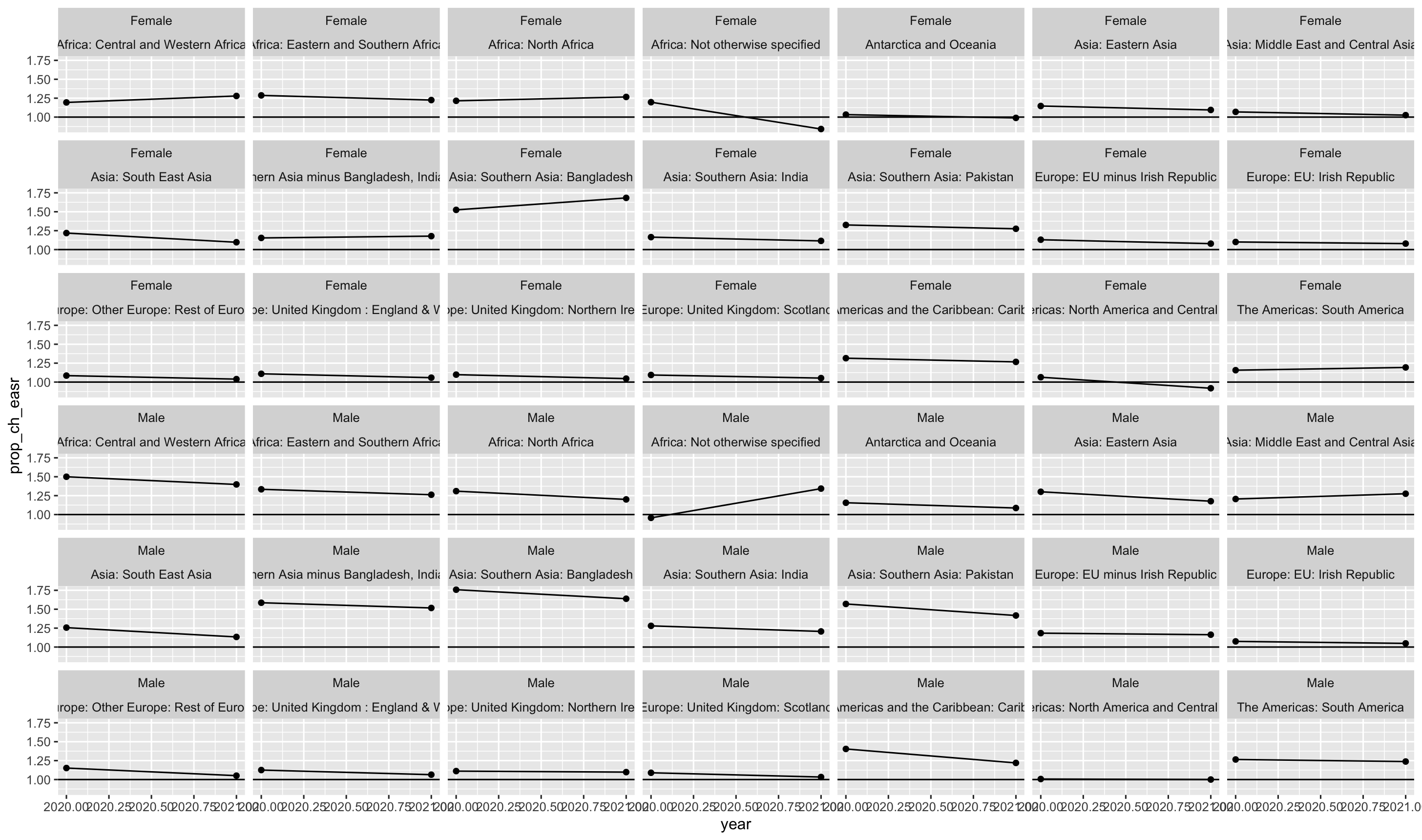


Figure S1: All-cause mortality EASR for 2020 and 2021 as a proportion of the 2019 values for males and females born in Bangladesh

## Annual Standardised Mortality Ratios by sex and country/region of birth, 2007-2021

### All-cause mortality 2007-2021, including COVID-19 in 2020 and 2021

To compare mortality in migrant populations to mortality of E&W, we present the SMRs for all-cause mortality *including* COVID-19 by the 19 country/regions of birth and sex over time from 2007 to 2021 in Figure S2, compared to the reference population of the corresponding sex born in E&W (SMR = 1; shown with black horizontal line). A SMR greater than 1 indicates higher mortality than the E&W born population, and a SMR less than 1 a lower mortality. 95% CIs are shown as shaded areas around the results.


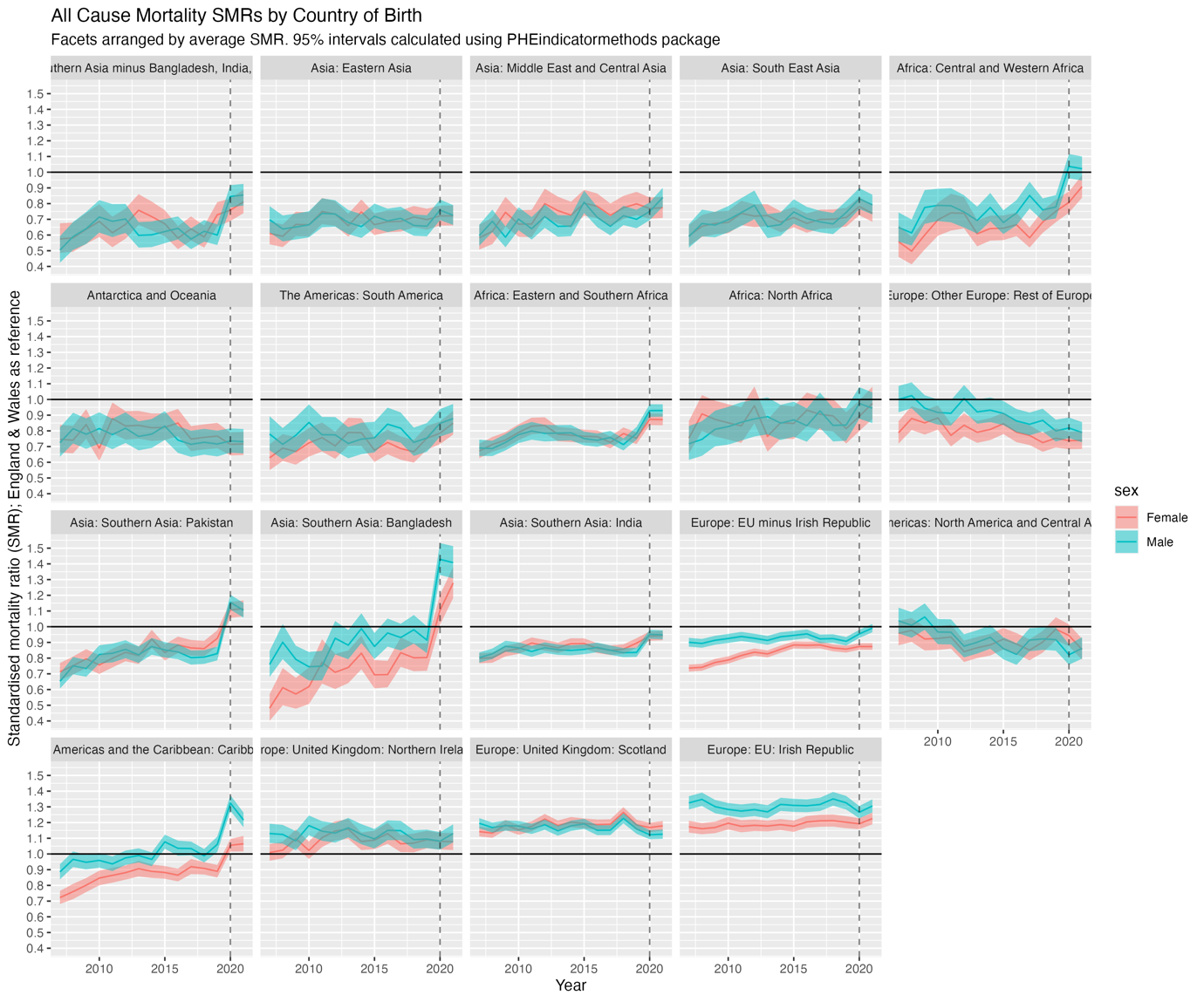


Figure S2: SMRs for all-cause mortality by sex and country/region of birth for migrants compared to non-migrants for the population of England and Wales, 2007-2021, with 95% confidence intervals (shaded). Note: horizontal black line at 1.0 represents the SMR of the reference population of males and females born in England and Wales.

## Regression of trends in SMRs: Supplementary results

The facet positions in Figure 3 show the relative mortality (dis)advantage of different migrant groups compared to the native-born population on average between 2007 and 2021. However, we also need to consider whether such relative (dis)advantages have been increasing or decreasing over time. We do this by presenting linear regressions lines of the SMR (dis)advantage over time. If the SMR is on average below 1, then upwards trend indicates this relative advantage has been decreasing or worsening over time; if the SMR is on average above 1, then downwards trends indicate the disadvantage has been decreasing over time; and so forth. Figure S3 shows the comparative SMRs of each of the 19 countries/regions of birth compared to the reference population, with these trend lines in SMRs added. We then present the results of the regression, by comparing that trend line to the E&W SMR over time as mean for 2007-2021. Table S1, S2 and Figure S3 show these results for the slope coefficient and whether they are statistically significant at the 5% level. However, without the mean SMR, these results are difficult to interpret as it is not clear whether there is a mortality advantage (SMR <1) that is improving/worsening; or a mortality disadvantage (SMR >1) that is improving/worsening. Thus, we present the mean SMR against the slope coefficient for each/country region and sex (Figure S3). These findings are summarised in Figure 5 in the main manuscript.

Table S1: Results of regression, males. Results to three decimal places.

Table S2: Results of regression, females. Results to three decimal places.

Figure S3 shows the results for the slope coefficient, with standard error represented by the width of the bar. Results significant at the 5% level i.e. with p value <=0.05 are shaded black, and those not significant in grey (see Table S1). A positive correlation and positive slope coefficient in Figure S3 indicate the health of that population is converging down towards that of non-migrants/E&W born, suggesting that the health advantage that may have been present in the past for migrants has reduced in more recent years. A negative correlation (negative slope coefficient) indicates that the population is diverging in all-cause mortality away from the E&W born non-migrant population, and this could be considered as the migrant mortality advantage increasing.


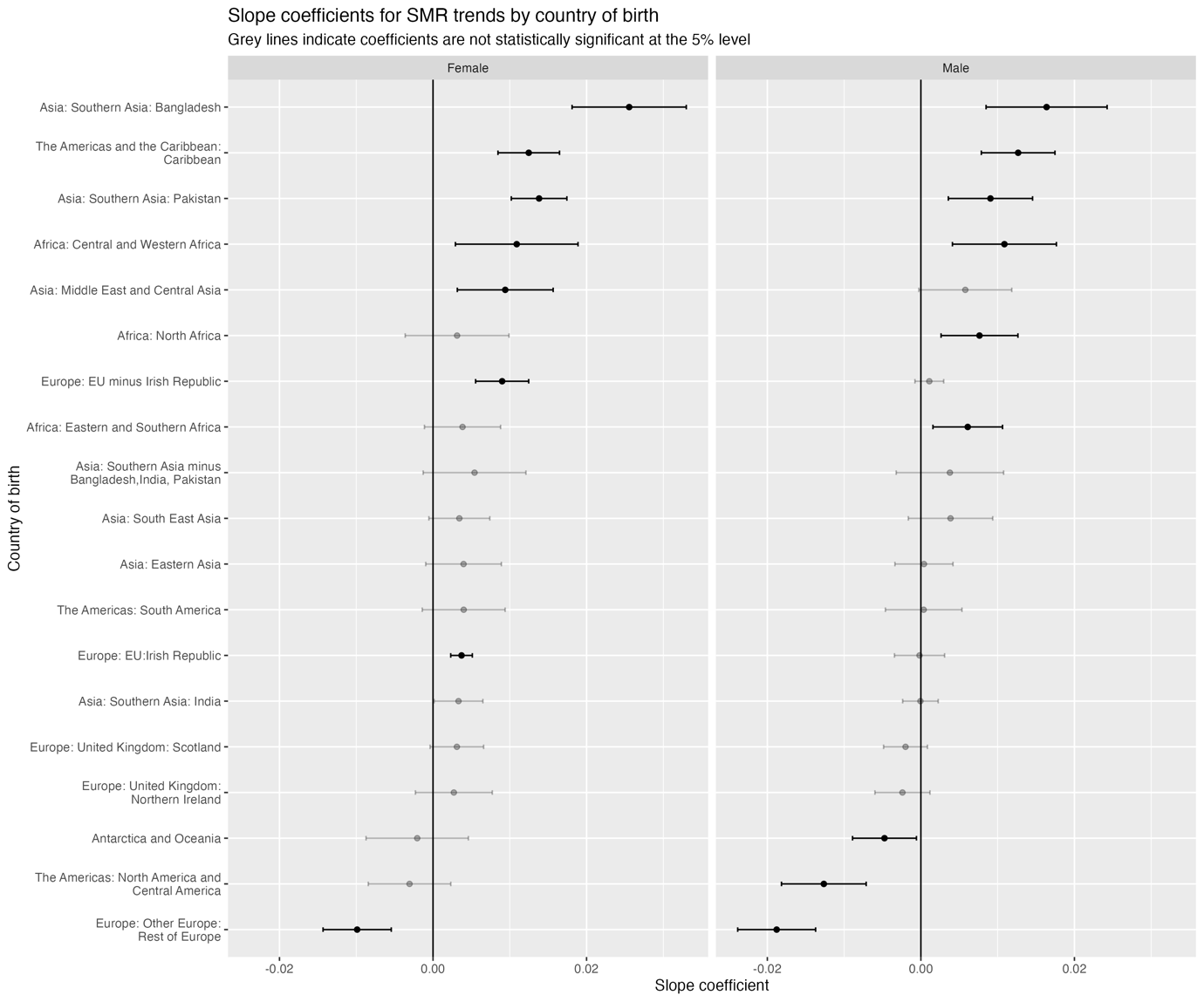


Figure S3: Slope coefficients for SMR trends by country/region of birth, population of England & Wales, 2007-2021 for all-cause mortality excluding COVID-19. Those results that are not significant at the 5% level i.e. that include 0 are shaded grey.


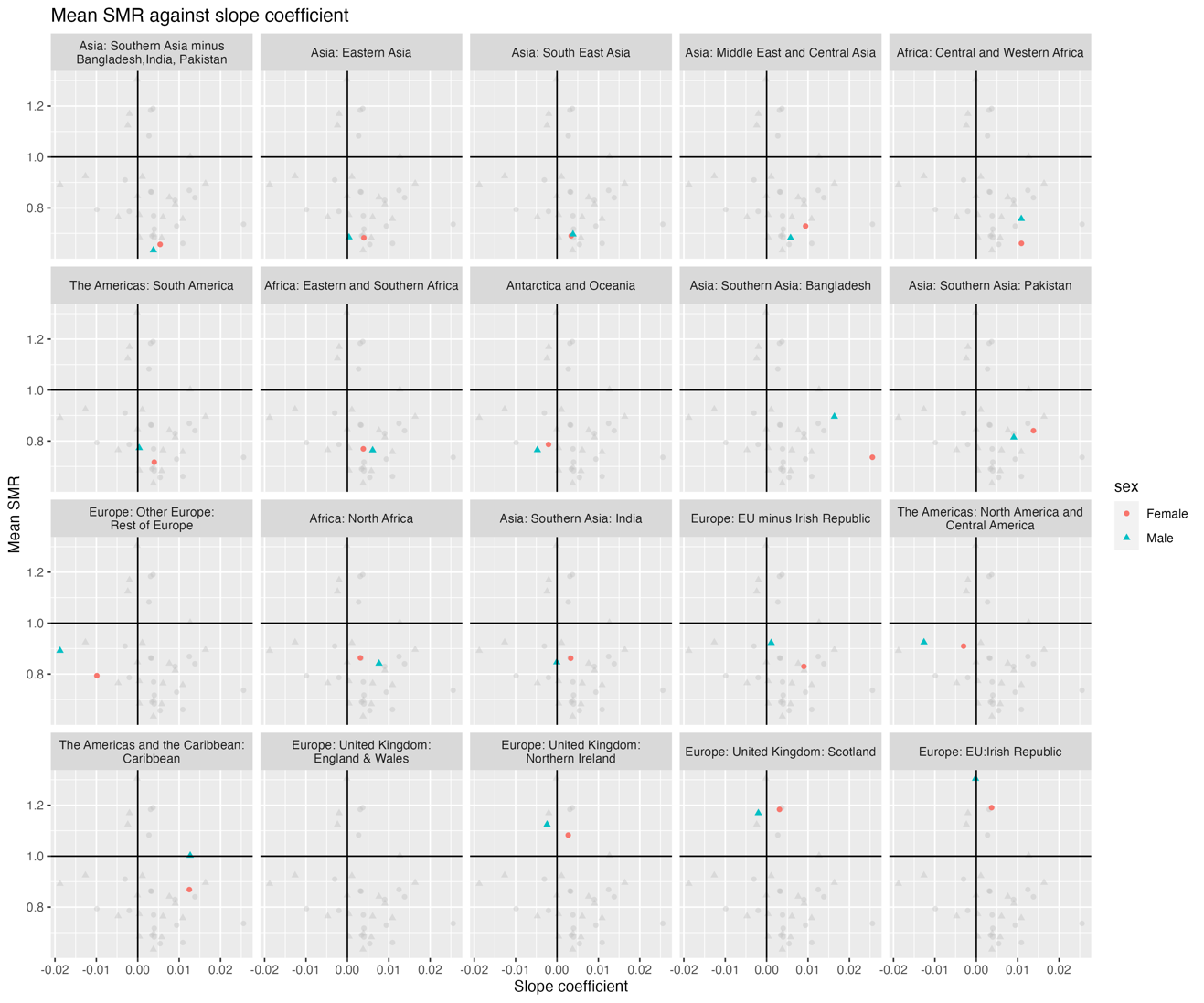


Figure S4: Mean SMR against slope coefficient for each country/region with England & Wales born as reference population; 2007-2021. For each subfigure, the blue and red dots represent the mean SMR and slope coefficient for male and females, respectively. The grey dots show the distribution of the other countries/regions of birth across the four quadrants. These four quadrants align with those shown in Figures 1 and 6 in the main manuscript.
